# Circulating Strains of Human Respiratory Syncytial Virus in Central and South America

**DOI:** 10.1371/journal.pone.0022111

**Published:** 2011-08-01

**Authors:** Merly Sovero, Josefina Garcia, Tadeusz Kochel, V. Alberto Laguna-Torres, Jorge Gomez, Wilson Chicaiza, Melvin Barrantes, Felix Sanchez, Mirna Jimenez, Guillermo Comach, Ivette Lorenzana de Rivera, Ana E. Arango, Roberto Agudo, Eric S. Halsey

**Affiliations:** 1 United States Naval Medical Research Unit 6, Lima, Peru; 2 Dirección General de Epidemiología, Ministerio de Salud, Lima, Perú; 3 Hospital Vozandes, Quito, Ecuador; 4 Hospital Solano, Buenos Aires, Argentina; 5 Hospital Infantil Manuel de Jesus Rivera, Managua, Nicaragua; 6 Hospital Nacional de Metapan, Metapan, El Salvador; 7 LARDIDEV-Biomed-UC, Maracay, Venezuela; 8 Universidad Nacional Autónoma de Honduras, Tegucigalpa, Honduras; 9 Universidad de Antioquia, Medellín, Colombia; 10 Dirección General de Epidemiología, Ministerio de Salud, Cochabamba, Bolivia; University of Georgia, United States of America

## Abstract

Human respiratory syncytial virus (HRSV) is a major cause of viral lower respiratory tract infections among infants and young children. HRSV strains vary genetically and antigenically and have been classified into two broad subgroups, A and B (HRSV-A and HRSV-B, respectively). To date, little is known about the circulating strains of HRSV in Latin America. We have evaluated the genetic diversity of 96 HRSV strains by sequencing a variable region of the G protein gene of isolates collected from 2007 to 2009 in Central and South America. Our results show the presence of the two antigenic subgroups of HRSV during this period with the majority belonging to the genotype HRSV-A2.

## Introduction

Human respiratory syncytial virus (HRSV) is a respiratory pathogen associated with substantial morbidity and mortality [1], infecting nearly all children in the first two years of life and hospitalizing 1% of those infected [2]. HRSV is one of the most contagious human pathogens, comparable to the measles virus. In one report the natural introduction of HRSV into a day-care setting resulted in infection of more than 90% of infants and children [3].

The HRSV genome encodes the synthesis of at least 11 viral proteins: three transmembrane glycoproteins known as the attachment glycoprotein (G), the fusion protein (F), and the small hydrophobic protein (SH); one matrix protein (M); two transcription factors (M1 and M2); three proteins associated with the nucleocapsid (N, P, and L); and two nonstructural proteins (NS1 and NS2) [4]. The F and G proteins are important antigenically because they stimulate the production of protective immune responses. Responses to the F protein include humoral and cytotoxic T-lymphocyte responses [5].

HRSV strains vary genetically and antigenically and have been classified into two broad subgroups:A and B (HRSV-A and HRSV-B, respectively) [6]–[9]. Analyses of the sequences of the N, P, SH, and G protein genes have confirmed the division of HRSV into these two subgroups and also have identified numerous genotypes or lineages within each subgroup [7], [8], [10]–[16]. Historically, the nomenclature assigned by Peret et al. [17], [18] and Venter et al. [19] for the different genotypes is: Protein Name-Subgroup-Variant.

The G protein is of particular interest because variability in this protein's corresponding nucleotide sequence is greater than in other proteins, both between and within the major antigenic subgroups of HRSV [15], [20], [21]. By analogy to the other paramyxoviruses and on the basis of experimental data, this protein is presumed to be the attachment protein of HRSV [4], and therefore, is very important, although not essential [22] for the replication of the virus. Variability in the G protein gene is concentrated in the extracellular domain, which consists of two hyper-variable regions separated by a central conserved region of 13 amino acids [8]. The second variable region, which corresponds to the C-terminal region of the G protein, reflects overall G protein gene variability and has been analyzed in molecular epidemiological studies [10], [17]. The significance of subgroup differentiation has been suggested by some studies. For example, HRSV-A may be more virulent than HRSV-B, and might result in greater disease severity among hospitalized infants [23]–[25].

Information exists about the presence of HRSV in Latin America [26]–[29] and a handful of reports describe the antigenic diversity of strains in Argentina and Chile [30], but little is known pertaining to the genetic diversity of this virus in the rest of the region. Studies from Argentina found more HRSV-A than HRSV-B during 2002 [31], [32] and no other data has been available since then.

In this study, we evaluated the genetic diversity of both HRSV-A and HRSV-B strains by sequencing a variable region of the G protein gene of isolates collected during a two and a half year period between January 2007 and June 2009 in Central and South America.

## Materials and Methods

### Ethics Statement

The views expressed in this article are those of the authors and do not necessarily reflect the official policy or position of the Department of the Navy, Department of Defense, nor the U.S. Government.

### Specimen Collection and Identification of HRSV Positive Samples

In collaboration with nine Central and South American countries (Nicaragua, Honduras, El Salvador, Venezuela, Colombia, Ecuador, Bolivia, Peru, and Argentina) that are part of a respiratory disease surveillance network, 15,875 pharyngeal swabs specimens were collected at hospitals from participants, regardless of age, with fever (≥38°C) and cough or sore throat between January 2007 and June 2009 [33]. Oropharyngeal swabs were collected at all sites except for the sites in Nicaragua were nasopharyngeal swabs collected. Swabs were transported in viral transport media on dry ice to the Naval Medical and Research Unit 6 (NAMRU-6) in Lima-Peru, where HRSV was identified by immunofluorescence-confirmed culture. Table 1: shows the number of samples from each country included in this study. Virus isolation was carried out by inoculation into four cell lines Madin-Darby canine kidney (MDCK), African green monkey kidney (Vero76 and VeroE6) and Rhesus monkey kidney (LLCMK2) cells (ATCC**,** Manassas, VA 20108)**.** Upon the appearance of a cytopathic effect (CPE) or after ten (or thirteen in the case of Vero cells) days of culture, the cells were spotted onto microscope slides. Cell suspensions were dried and fixed in chilled acetone for 15 minutes. Immunofluorescence assay was performed to identify the virus isolates from cell culture, using a direct fluorescence assay (DFA). The Respiratory Virus Screening and Identification Kit (D3 DFA Respiratory Virus Diagnostic Hybrids; Athens, OH) was utilized for the identification of adenoviruses, influenza A virus, influenza B virus, para-influenza viruses (types 1, 2, and 3), and HRSV. HRSV positive isolates obtained by DFA were required for strain characterization.

**Table 1:** Number of Samples by Country.

Country	Total Samples	HRSV Positive	Sequenced
**Argentina**	845	20	**15**
**Bolivia**	477	10	**3**
**Colombia**	859	17	**2**
**Ecuador**	1,161	14	**4**
**El Salvador**	316	4	**1**
**Honduras**	621	5	**-**
**Nicaragua**	1167	94	**32**
**Peru**	9,916	130	**37**
**Venezuela**	514	2	**2**
**Total**	**15,875**	**296**	**96**

The number of total participants, HRSV-positive samples by immunofluorescence, and the sequenced samples for each country.

### Ethics

The study protocol was approved by the Naval Medical Research Center Institutional Review Board (Protocol NMRCD.2002.0019) in compliance with all applicable federal regulations governing the protection of human subjects. All participants underwent a verbal consent process. No informed consent document was prepared since samples were obtained through procedures considered less than minimal risk by the aforementioned institutional review board.

### RNA Extraction and RT-PCR

Viral RNA extraction was performed in a biosafety level-3 laboratory with enhanced containment practices. Nucleic acid was extracted with the use of viral RNA kit (QIAamp, Qiagen®) from 140 µl of the naso/oropharyngeal swabs and was amplified in a reverse transcriptase PCR (RT-PCR). RT-PCR was performed by using a SuperScript III One-Step RT-PCR System (Invitrogen; San Diego, CA); 5 µl of extracted RNA was added to a master mixture composed of an enzyme mixture (SuperScript III RT/Platinum *Taq*), 3.2 mM MgSO4, and 0.4 mM dNTP. The G gene primers were SHA (TCGAGTCAACACATAGCATTC) and F1 (CAACTCCATTGTTATTTGCC) [18]. This primer pair was used at 20 µM in the amplification.

Five microliters of RNA were added to the 20 µl RT-PCR master mixture. The amplification was carried out in a thermocycler 7700 (Applied Bioystems; Foster City, CA). Cycling conditions included a reverse transcription step at 50°C for 30 minutes and 94°C for 2 minutes followed by 40 PCR cycles: 94°C for 30 seconds; 55°C for 30 seconds; 72°C for 1 minute, and final incubation at 72°C for 7 minutes. The amplified product of ≈1200 bp was analyzed by electrophoresis on a 2% agarose gel.

### Phylogenetic Analysis

Phylogenetic trees were constructed on the basis of the G gene sequence from 96 samples. For direct sequencing of viral nucleic acids from HRSV culture-positive specimens, gene fragments were amplified and sequenced with the use of the Big Dye terminator cycle sequencing kit (version 3.1, Applied Biosystems) on a Genetic Analyzer system (version 3130xL, Applied Biosystems). Gene sequences were assembled aligned and edited using Sequencher and BioEdit (version 7.0.0 -Isis Pharmaceuticals, Inc.) software.

All samples were classified into two distinct subgroups, named HRSV-A and HRSV-B. The nucleotide sequences obtained correspond to the extracellular region of the G protein, from amino acid (aa) 24 to 221. This section includes the first variable region of the G protein, the 13 aa conserved region in position 164-176 and a part of the second variable region of this protein. The sequences were analyzed and aligned with the available HRSV sequences from the GenBank database by using the CLUSTAL X program (version 1.83). Phylogenetic trees were constructed by the neighbor-joining method in MEGA software (version 3). The statistical significance of the tree topology was tested by bootstrapping (1,000 replicas). Pairwise distances between and within the genotypes at the nucleotide level were calculated with Kimura 2 parameters and with Poisson correction at the amino acid level with MEGA software.

## Results

**Figure 1:**
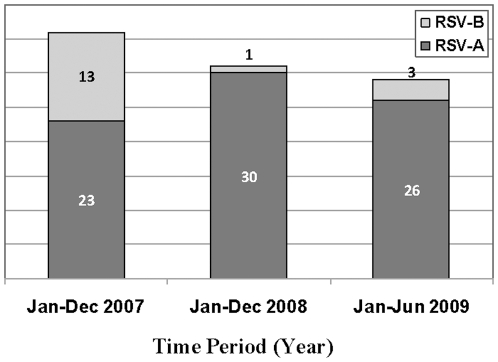
HRSV subgroups by Year. The number of samples for each HRSV subgroup (A and B) per year. For years 2007 and 2008, the month of collection are from January to December. For year 2009, the months of collection are from January to June.

Through the respiratory surveillance network, 15,875 samples were collected from different countries in Central and South America (Table 1). From those, 296 HRSV positive samples were detected using DFA after cell culture was performed (Figure 1). In agreement with previously published studies, 95% of HRSV positive samples came from participants younger than 15 years old with an average age of 1.2 years, and only 14 samples belonged to adults older than 20 years old. Participants were 45% (n = 133) male and 55% (n = 163) female, and this small difference was not statistically significant.

Among the HRSV-infected patients, 100% presented with cough and rhinorrhea, 46% had malaise, 37% experienced tearing, 30% had sore throat, and 28% had wheezing. A total of 67 participants (22.6%) were hospitalized and 28 (9.5%) were diagnosed with pneumonia. From the 79 participants infected with HRSV-A, 18 (22.8%) were hospitalized. Conversely, none of the participants infected with HRSV-B were hospitalized. Less common symptoms included headache, eye pain, and muscular pain.

By immunofluorescence, we detected up to 19 co-infections (6.4% of HRSV positive samples). The most common co-infecting pathogens were adenovirus (n = 7) and influenza A virus (n = 6). Co-infections with influenza B virus (n = 2), HSV (n = 2), parainfluenza 3 virus (n = 1) and echovirus (n = 1) were also detected.

From the 296 HRSV infections found, 96 HRSV were selected randomly to be analyzed by sequencing. Nucleotide analysis showed that both HRSV subgroups (A and B) circulated in Latin America during the period of study (Figure 1). The obtained sequences are available online in Genbank with accession numbers: JF905487-JF905560.

**Figure 2:**
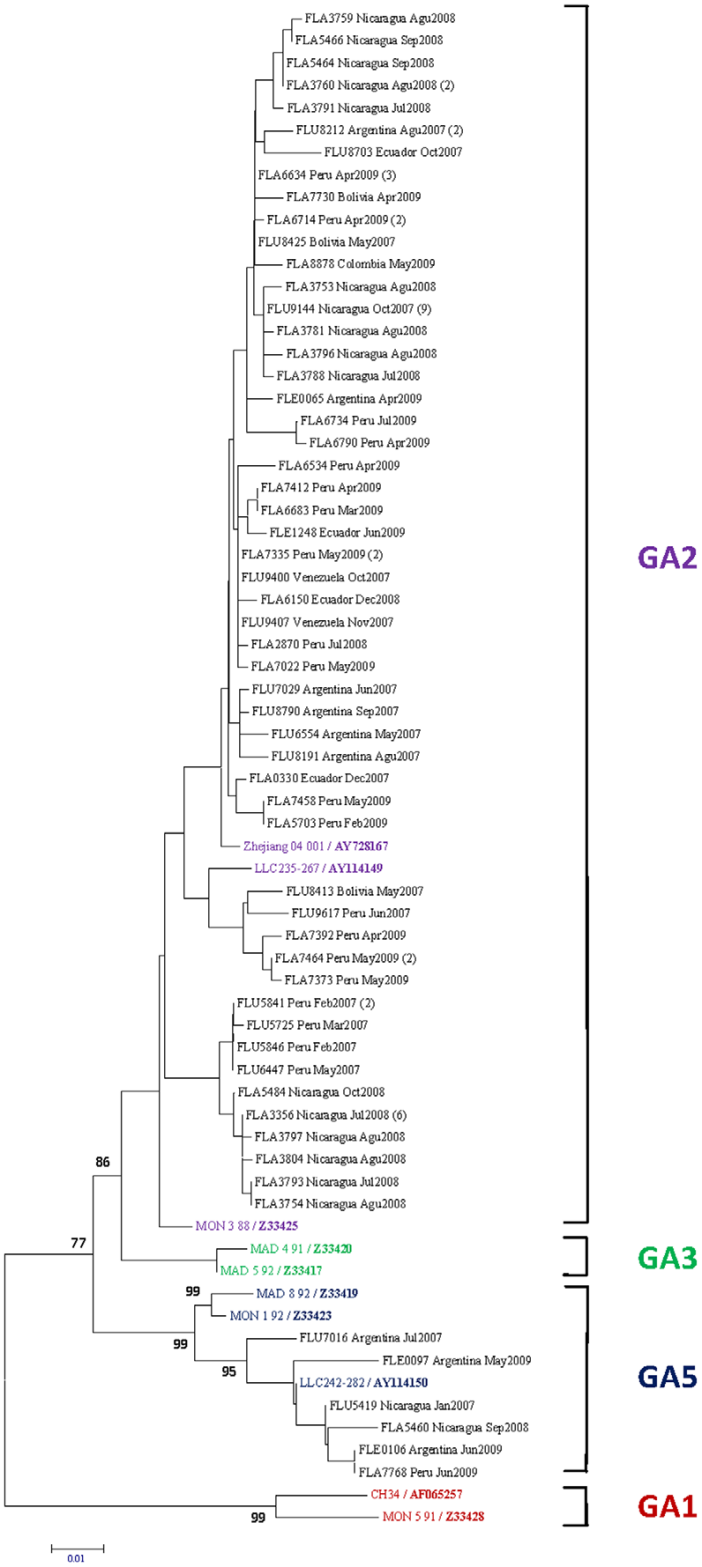
HRSV subgroup A Phylogenetic Tree. 601 nucleotides of the G protein gene were amplified, sequenced, and compared to published sequences from GenBank. We have labeled the samples according to the following format: “Sample code/Country of collection/Month- Year of collection”. The number of strains with identical sequences is shown in parenthesis to the right of one representative strain. The comparison sequences are complete genome sequences from GenBank, these are presented in the following format: “Strain Number/GenBank Accession Number in Bold”. Nucleotide sequences were aligned by using Clustal X. Phylogenetic analyses were performed using the Kimura two-parameter model as a model of nucleotide substitution and using the neighbor-joining method to reconstruct phylogenetic trees (MEGA version 2.1).

**Figure 3:**
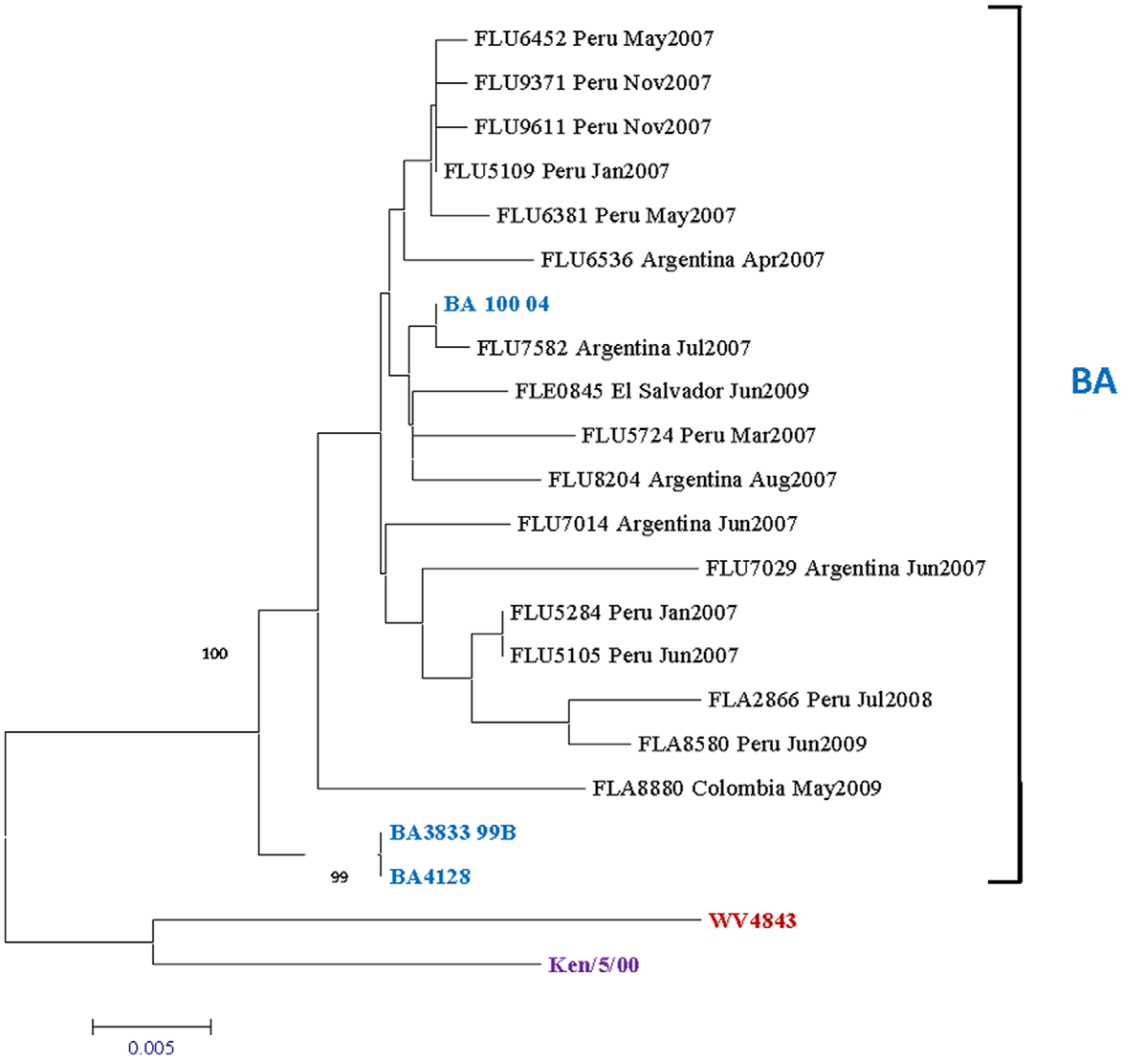
HRSV subgroup B Phylogenetic Tree. 630 nucleotides of the variable region of G protein gene were amplified, sequenced, and compared to published sequences from GenBank. Labeling and analysis were performed as in Figure 2.

Phylogenetic trees in Figure 2 and 3- show the diversity of the samples upon analysis of their G protein gene nucleotide sequence of HRSVA and -B respectively. The 79 HRSV-A samples (82.3% of HRSV-positive samples) grouped clearly into two clades, GA2 and GA5, with GA2 accounting for 92% (n = 73) of the samples (Figure 2). In respect to the HRSV-B subgroup, the 17 samples (17.7% of HRSV positive samples) grouped into only one clade belonging to the BA [34] genotype (Figure 3).

## Discussion

In this study, we investigated the genetic characteristics of circulating HRSV virus in the regions of Central and South America from January 2007 until June 2009, a time period prior to the introduction of pandemic influenza. We detected 296 HRSV cases by DFA accounting for 1.8% of all respiratory samples received from the nine countries. There are many reports on the different methods to detect HRSV [35]–[38], and the authors are aware that immunofluorescence on cultured cells might not be the most powerful technique to accomplish this, and therefore no frequencies or proportions of HRSV infection will be discussed in this manuscript. The aim of this study was to molecularly analyze the circulating strains of HRSV in the region. Future studies utilizing direct PCR may better address questions of frequency of infection with this virus.

However, it was clear that the most affected population by HRSV infection was children, in particular, children less than one year old. We also showed that co-infection with other viruses is not rare (6.4% of HRSV positive samples). Our findings differ from a report of an outbreak of para-influenza 3 and HRSV [39] in that we detected only one co-infection with these two viruses. Influenza A and adenovirus were the two most common co-infections detected in our patients with HRSV.

It is important to note that the proportion of hospitalization among HRSV-infected patients was elevated compared to the proportion among all recruited participants with ILI symptoms (22.6% vs 5.1% with a p-value <0.01). In agreement to what others reported [23]–[25], we found that HRSV-A resulted in greater disease severity than HRSV-B as shown by the hospitalization rate of 22.8% for patients infected with HRSV-A and zero for patients infected with HRSV-B. This difference is statistically significant with a p-value <0.05. Our results reveal the presence of both HRSV-A and HRSV-B, as previously reported in Argentina and Brazil [28], [31], [32].

Genetic variability was evident HRSV-A strains, with two main sub-branches identified, corresponding to genotypes GA2 and GA5. Our phylogenetic analysis revealed that GA2 was predominant throughout the region compared to GA5 in the analyzed strains.

In respect to HRSV-B, we were able to identified only one strain featuring a 60-nucleotide insertion in the second variable region of G protein, a BA-like strain. The BA genotype (named for Buenos Aires [34]) was detected in Japan in 2003 [40] and subsequently has been noted in China [41], Thailand [42], Japan [43], South Africa [44], and India [21], becoming the predominant HRSV-B genotype in circulation [45].

Together, these data provide a better understanding of the HRSV strains in circulation in Latin America and show that HRSV strains appear to be similarly distributed worldwide [20], [21], [40], [42]–[46].
